# The Changes of Heart miR-1 and miR-133 Expressions following Physiological Hypertrophy Due to Endurance Training 

**DOI:** 10.22074/cellj.2020.7014

**Published:** 2020-09-08

**Authors:** Mohammad Fathi, Reza Gharakhanlou, Razieh Rezaei

**Affiliations:** 1 Department of Physical Education and Sport Sciences, Faculty of Humanities Sciences, Lorestan University, Khorramabad, Iran; 2 Department of Physical Education and Sport Sciences, Faculty of Humanities Sciences, Tarbiyat Modares University, Tehran, Iran; 3 Faculty of Physical Education and Sport Sciences, Shahid Chamran University of Ahvaz, Ahvaz, Iran

**Keywords:** Endurance Training, Hand2, Mef2c, miR-1, miR-133

## Abstract

**Objective:**

MicroRNAs (miRNAs) play a key role in the development of the heart. Recent studies have shown that miR- 1 and miR-133 are key regulators of cardiac hypertrophy. Therefore, we aimed to evaluate the effect of an endurance training (ET) program on the expressions of these miRNAs and their transcriptional network.

**Materials and Methods:**

In this experimental study, cardiac hypertrophy was induced by 14 weeks of ET for 1 hour per day, 6 days per week at 75% VO2 max). The rats (221 ± 23 g) in the experimental (n=7) and control (n=7) groups were anesthetized to evaluate heart morphology changes by echocardiography. Next, we evaluated expressions of miR-1 and miR-133, and heart and neural crest derivatives express 2 (Hand2), Mef2c, histone deacetylase 4 (Hdac4) and serum response factor (Srf) gene expressions by real-time polymerase chain reaction (PCR). Finally, the collected data were evaluated by the independent t test to determine differences between the groups

**Results:**

The echocardiography result confirmed physiological hypertrophy in the experimental group that underwent ET as shown by the increased left ventricular weight/body surface area (LVW/BSA) (P=0.004), LVW/body weight (BW) (P=0.011), left ventricular diameter end-diastolic (LVDd) (P=0.003), and improvements in heart functional indexes such as fractional shortness (FS) (P=0.036) and stroke volume (SV) (P=0.002). There were significant increases in the expressions of miR-1 (P=0.001) and miR-133 (P=0.004). The expressions of Srf, Hdac4, and Hand2 genes significantly increased (P<0.001) in the experimental group Compared with the control group. The expression of Mef2c did not significantly change.

**Conclusion:**

The expressions of miR-1 and miR-133 and their target genes appeared to be involved in physiological hypertrophy induced by ET in these rats.

## Introduction

In recent years, microRNAs (miRNAs) are considered one of the most important factors involved in myocellular processes, including cell differentiation, proliferation, heart disease, and muscle adaptation (1). Among the known miRNAs, myomiRs (special muscle miRs) are specifically expressed in skeletal and cardiac muscle tissues (2). A wide array of studies have focused on myomiRs functions (3, 4). myomiRs have important roles in myogenesis, embryonic muscle growth, and cardiac function and hypertrophy (5). In a review article, van Rooij et al. (2) introduced a network of transcription factors involved in the regulation of ventricular growth and cardiomyocyte differentiation of the heart, in which myomiRs play a central role. Among the myomiRs, miR-1 and miR-133 are expressed in striated muscle and have different roles. While miR-1 promotes myogenesis by targeting histone deacetylase 4 (*Hdac4*), a transcriptional repressor of muscle gene expression, miR-133 enhances myoblast proliferation by repressing serum response factor (*Srf*) (6).

*Srf* is a member of the MADS-box family of transcription factors and an important regulator of various genes that are necessary for cardiac function and development. Srfdependent genes induce some contractile proteins such as skeletal α-actin, β myosin heavy chain (βMHC), cardiac α-actin, myosin light chain-2 (MLC-2v), and dystrophin. The importance of the Srf protein in regulating both contractile protein genes and genes that encode proteins involved in regulating the action of the contractile apparatus suggest that Srf exerts control over multiple aspects of cardiac function (7). Heart and neural crest derivatives express 2 (*Hand2*), a transcription factor that promotes ventricular cardiomyocyte expansion, is a target of miR-1 (8). It has been shown that the *Mef2c* transcription factor, an essential regulator of muscle development, directly activates transcription of a bicistronic primary transcript that encodes miR-1 and miR-133 via an intragenic muscle-specific enhancer located between the miR-1 and miR-133 coding regions (9). As van Roiij et al. (2) showed, the all of mentioned factors is in a transcriptional network that functions in cardiomyocyte proliferation and differentiation, control of cardiac growth, and conductance.

In addition to miRNAs, physical activity, especially endurance activities, causes changes in heart tissue (10) that lead to structural changes and changes in cardiac function such as left ventricle (LV) hypertrophy (physiology hypertrophy) (11), which coincides with an increase in stroke volume (SV), ejection fraction percent (EF%), and LV mass (12) of the heart.

Thus, the effect of long-term endurance training (ET) on miRNAs and their upstream (*Srf* and *Mef2c*) and downstream (*Hdac4 and Hand2*) genes (2) in the heart is intriguing. In the field of exercise and cardiac adaptation, few studies have evaluated the effect of ET on expression of miR-1 and miR-133 in a hypertrophied heart (13). No data has assessed both functional and structural heart changes and simultaneously measured miR-1 and miR- 133 transcription network changes involved in cardiac hypertrophy, which is very important in cardiomyocyte proliferation and differentiation. Given the probable changes in cardiac remodelling by ET, this study assessed whether 14 weeks of ET would change the expressions of miR-1 and miR-133 and their upstream and downstream genes, and cause hypertrophy of the heart tissue in rats.

## Materials and Methods

**Fig.1:**
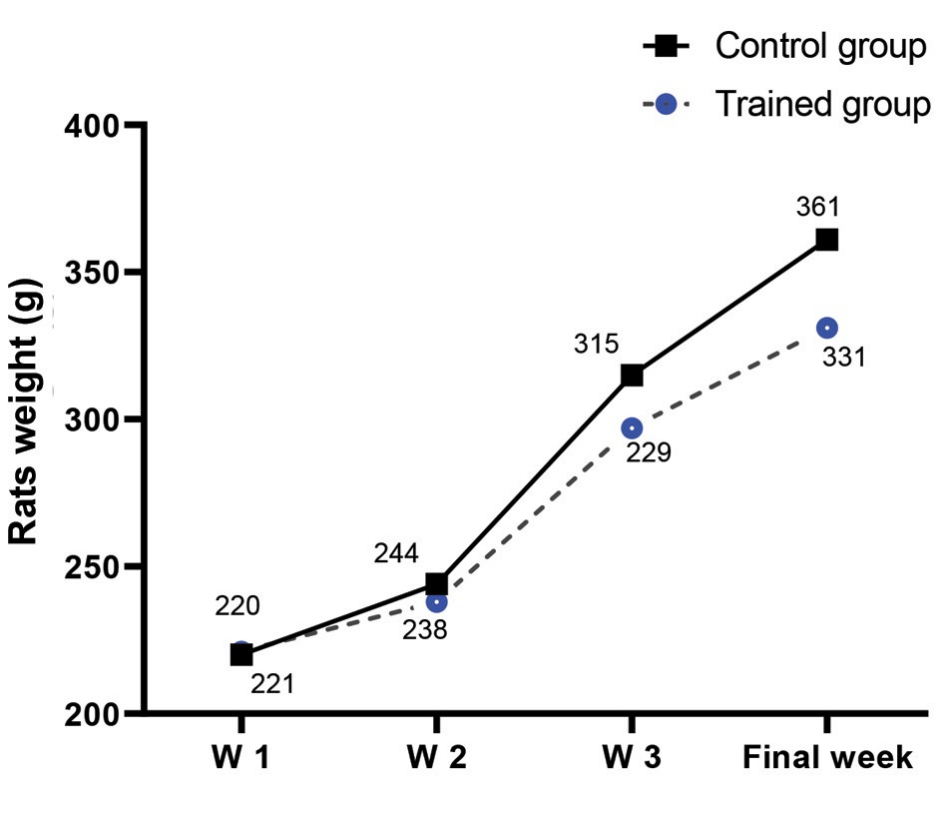
The weight trend (means) in the rats during 14 weeks of endurance training. W; Week.

This experimental investigation was conducted at Tarbiat Modares University, Tehran, Iran. A total of 14 healthy male adult Wistar rats, 10 weeks of age, that weighed between 175 and 200 g (Fig .1) were purchased from Pasteur Institute, Tehran, Iran. The rats were maintained under the following conditions: 12-hour dark (7 p.m. to 7 a.m.)/12-hour light cycle (7 a.m. to7 p.m.), 23 ± 2°C, and 30- 70% humidity to enable them to acclimate to the laboratory environment. The rats had free access to water and food, which was purchased from Behparvar Animal Chow Company, Iran. The rats were weighed before the start of the protocol. The experimental approved by the Ethics Committee of the Iran National Science Foundation (code: 90003724). Every attempt was made to decrease the number of animals used and their suffering. All of the observations were performed by a single individual. The rats were randomly assigned by simple randomization to a control (CON) group (n=7) and experimental (ET) group (n=7). The ET group was subjected to a prolonged ET program.

### Training protocol

After a one-week acclimation period (9 m/minute, 10 minute/day, 4 days a week). The rats were weighed and marked. Rats in the ET group were trained by running on a level motorized rodent treadmill, 6 days per week for 14 weeks. At the start of each session, the rats were allowed to warm up by running for 5 minutes at 12 m/minute. After completion of the warm up period, the main exercise was begun. During the first 6 weeks, the speed of the treadmill and duration of the training sessions were gradually increased, as follows: week 1 (20 m/minute), week 2 (22 m/minute), week 3 (25 m/minute), week 4 (27 m/minute), week 5 (29 m/minute), and week 6 (30 m/minute). On the first day, the exercise duration was 12 minutes, which was increased daily by approximately more than 2 minutes per day until it reached 50 minutes at the end of the third week. We added an incline beginning with the seventh week, which reached 5 degrees by the end of the tenth week. This protocol was maintained until the end of the fourteenth week. According to a study by Wisloff et al. (14) the intensity of this protocol was equal to 75% VO2max in the rats. At the end of the main exercise period, the rats were allowed to cool down at a speed of 9 m/ minute for 5 minutes. The endurance protocol was performed between 5-7 pm each day. The measurements were obtained 48 hours after the end of the last training session. With the exception of the training protocol, the other conditions were similar to the CON group.

### Echocardiography recording

**Fig.2:**
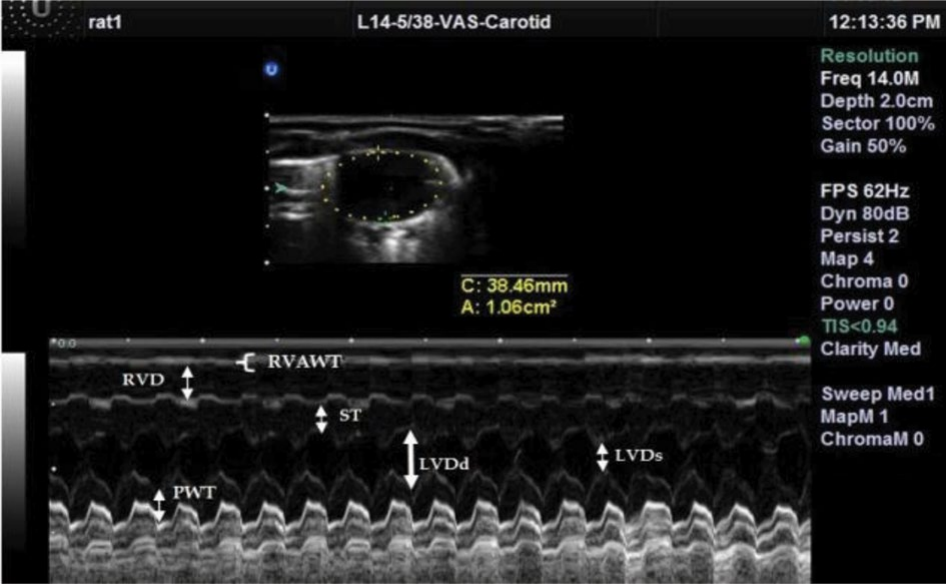
Two-dimensional targeted M-mode echocardiograms from the heart of one rat from the endurance-trained (ET) group. RVAWT; Right ventricular anterior wall thickness, RVD; Right ventricular dimension, ST; Septum thickness, LVDd; Left ventricular diameter diastole, LVDs; Left ventricular diameter diastole, and PWT; Posterior wall thickness.

The rats from both groups were administered a light anaesthesia using ketamine and xylazine, and placed in the supine position. Ultrasound gel was placed on the thorax of each rat to enable optimal visibility. The echocardiography was performed according to the guidelines of the American Society of Echocardiography (15). We used an ultrasound machine (Sonix Touch Ultrasound System, Ultrasonix Medical Corp., Richmond, ON, Canada) and a 14 MHz linear array transducer. Images were obtained with the transducer placed on each animal’s shaved chest. The animals were scanned from below at a depth of 2 cm with the focus optimized at 1 cm. Wall thickness and LV dimensions were obtained from a short axis view at the level of the papillary muscles (Fig .2). Echocardiography was completed within 10 to 15 minutes. The fractional shortness percent (FS%) was calculated using the following equation:

(LVDd-LVDs/LVDd)×100

where: LVDd is the left ventricular diameter enddiastolic.

The ejection fraction (EF) was calculated by the following equation:

(LVEDV-LVESV/LVEDV)×100

where: LVDd and LVDs=LV end-diastolic/systolic diameter and LVEDV and LVESV=LV end-diastolic/systolic volume (16).

The left ventricular end-systolic/diastolic volume was calculated by the Teichholz formula:

LVESV=[7.0/(2.4+LVSd)]×LVSd^3^

and

LVEDV=[7.0/(2.4+LVDd)]×LVDd^3^

SV and FS were also calculated as the measured differences between the LVEDV and LVESV and [LVDd- LVDs]/LVDd, respectively (17).

### Tissue preparation and other measurements

One day following the echocardiographic examination, the animals were weighed first, and subsequently injected with an overdose of ketamine (90 mg/kg) and xylazine (10 mg/kg). The animals were sacrificed by administration of overdoses of ketamine and xylazine 48 hours after the last session. We calculated the body surface area (BSA) of each animal by measuring their lengths (mouth to tail root) while they were unconscious, and then we removed the heart and tibia bone. The heart was rapidly and carefully excised. The dissected heart and left ventricle (including the septum) were weighed to four decimal digits. We measured the tibia length by using a calliper, heart and left ventricular weight (LVW) to normalization, and evaluated the hearts for hypertrophy. After the measurement of LVW and heart weight, the tissue samples of LV and heart were quickly frozen in liquid nitrogen and stored at -80°C until needed. Tibia length, BSA and body weight (BW) were used to normalize the heart and LVW changes. We used the Excel progam to calculate compounded parameters such as EF, FS, and other indexes.

BSA=6.67×W^0.7^×[0.34/ (∛ W/L)] (18)

### RNA isolation and cDNA synthesis

Total RNA was isolated from the frozen left ventricles by using TRIzol (Invitrogen, Inc.) and chloroform (Merck, Germany) according to the manufacturer’s instructions. Briefly, the LV extracted tissue was pulverized in liquid nitrogen by a mortar and pestle, and the pulverized sample was transferred to TRIzol 1ml. The final product was centrifuged at 12000 × g for 10 minutes at 4°C. Then, the samples were mixed with chloroform in 1:5 portions and shaken vigorously for 15 seconds. The supernatant was centrifuged at 12000 × g for 10 minutes at 4˚C and the supernatant were removed. Finally, the portion that contained RNA was removed and mixed with isopropanol in 1:5 portions, allowed to remain for 10 minutes at room temperature, and then centrifuged at 12000 × g at 4˚C for 10 minutes. The RNA was washed and dissolved in 20 μL RNase-free water. RNA purity was determined by UV spectrophotometry (Eppendorf, Germany) at 260 nanometre: RNA purity was determined by calculating the absorbance ratio at 260 and 280 nm. GelRed staining was used to confirm RNA purity. Purification was accepted when the 260/280-nm absorbance ratio was above 1.8. Isolated RNA was stored at -80˚C. Total RNA was converted to cDNA using the Revert Aid First Strand cDNA Synthesis Kit (Thermo Scientific, Waltham, MA, USA) according to the manufacturer’s instructions.

### Real-time polymerase chain reaction

Real-time polymerase chain reaction (PCR) was performed using the Takara Master Mix (RR820A SYBR® Premix Ex Taq™ II. Tli RNase H Plus) for determining mRNA expression levels of *Hand2, Mef2c, Srf,* and *Hdac4* (Step One Plus™ Real-Time PCR Systems, Applied Biosystem America). The reaction mixture was performed in final volume in 20 μL that included 10 μL of Syber Green, 1 μL of forward primer, 1 μL of reverse primer, 1 μL of cDNA, and 7 μL of DEPC water. Each reaction was run in triplicate. GeneRunner software and NCBI (http://www.ncbi.nlm.nih.gov/tools/primerblast/), respectively, were used to design and BLAST of the *Hand2, Mef2c, Srf, Hdac4* and glyceraldehyde-3-phosphate dehydrogenase (*Gapdh*) primers according to the NCBI Gene Bank. Table S1 (See Supplementary Online Information at www.celljournal.org) lists the primer sequences used in this experiment. The thermal program used in real-time PCR was 95°C for 30 seconds, 95°C for 5 seconds, and 60°C for 30 seconds (40 cycle repetitions). The standard and melting curves were drawn and analysed for optimization of the experiment and assessment of data accuracy, respectively. *Hand2, Mef2c, Srf* and* Hdac4* mRNA expressions were normalized using *Gapdh* as the housekeeping gene.

### MicroRNA detection

We measured the amount of miR-1 expression with the 5ˊ-UGGAAUGUAAAGAAGUAUGUAU- 3ˊ sequence and miR-133 expression with the 5ˊ-UUUGGUCCCCUUCAACCAGCUG-3ˊ sequence. The reagents for cDNA synthesis (#203300), SYBR Green master mix (#203450), primers miR-1 and miR-133, and U6 (small nuclear RNA as miRNAs housekeeping) were purchased from Exiqon (Vedbaek, Denmark). cDNA synthesis and real-time PCR for miR-1 and miR-133 were done according to the manufacturer’s protocols as described in our previous study (19). The equipment used in this experiment were calibrated regularly.

### Statistical analysis

All data are presented as mean ± standard deviation (SD). We confirmed that all continuous variables were normally distributed using the Shapiro-Wilk test and the equality of variances using Levene’s test. Next, the independent t-test was used to assess the main effects of the ET prog that included changes in mRNA expressions of miR-1 and miR-133, and echocardiographic indexes after the ET program. All analyses were performed using the SPSS statistical software (version 20,SPSS Inc, Chicago, IL, USA) The significance level was set at P<0.05. The graphs were reported based on mean ± SD.

**Table 1:** Normalized heart and left ventricle based BSA and tibia length of rats (n=14) and BW, HW, tibia length, body surface, and cardiac structural and functional indexes of rats’ hearts after 14 weeks of endurance training


Index	Group	Mean ± SD	P value

LVW (g)/tibial length (c)	ET	0.01913 ± 0.00138	0.216
	CON	0.01826 ± 0.00109	
LVW (g)/BSA (cm^2^)	ET	0.168 ±0 .0086	0.004^**^
	CON	0.15 ± 0.0068	
LVW (g)/HW (g)	ET	0.6136 ± 2.73	0.096
	CON	0.6465 ± 3.85	
LVW (g)/BW (kg)	ET	2.3 ± 0.18	0.011^*^
	CON	2.05 ± 0.12	
LVW (g)	ET	0.75918 ± 0.04904	0.435
	CON	0.73968 ± 0.04472	
BSA (cm^2^)	ET	451.19 ± 22.7	0.021^*^
	CON	481.57 ± 20	
HW (g)	ET	1.23 ± 0.059	0.058
	CON	1.14 ± 0.096	
Final BW (g)	ET	331.2 ± 33.4	0.065
	CON	361.2 ± 16.8	
Tibia length (mm)	ET	39.7 ± 0.95	0.476
	CON	40.57 ± 2.86	
Septum (mm)	ET	1.96 ± 0.499	0.323
	CON	1.7 ± 0.203	
LVDd (mm)	ET	5.001 ± 0.719	0.003^**^
	CON	3.98 ± 0.13	
RVDd (mm)	ET	1.81 ± 0.47	0.812
	CON	1.77 ± 0.158	
RVAWT (mm)	ET	1.21 ± 0.2	0.379
	CON	1.29 ± 0.11	
RVD (mm)	ET	1.77 ± 0.4	0.841
	CON	1.73 ± 0.32	
PWT (mm)	ET	1.57 ± 0.437	0.939
	CON	1.585 ± 0.166	
FS (%)	ET	63.84 ± 8	0.036^*^
	CON	55.41 ± 4.9	
EF (%)	ET	91.02 ± 4.8	0.066
	CON	86.1 ± 4.1	
SV (ml)	ET	3.18 ± 0.53	0.002^**^
	CON	2.2 ± 0.24	


LVW; Left ventricular weight, BSA; Body surface area, EF; Ejection fraction, SV; Stroke volume, HW; Heart weight, BW; Body weight, LVDd; Left ventricular diameter end-systolic, RVDd; Right ventricular diameter end-systolic, RVAWT; Right ventricular anterior wall thickness, RVD; Right ventricular dimension, PWT; Posterior wall thickness, FS; Fractional shortness, ET; Endurance training, and CON; Control.

## Results

Table 1 and Figure 1 list the rat’s morphology characteristics that included BW, HW, LVW, tibia length, and body length. There was no significant difference between the groups in BW at the start of the protocol (P<0.801). BW of the ET group rats did not change compared with CON group rats at the end of the protocol (P≤ 0.063, Fig .1).

Other studies have used parameters such as tibia length (20) and BSA (18) to normalize heart hypertrophy. Therefore, we calculated these indexes (Table 1) and noted between the two groups in mean HW/BSA and mean BH/BW ratios at significance level of P<0.01.

In the normalized parameter, there was a difference between LVW/BSA (P=0.004) and LV/BW (P=0.011). Table 2 lists the mean echocardiographic parameters after 14 weeks of endurance exercise. There were significant differences between the two groups in LVDd (P=0.003) and FS (P=0.036).

**Fig.3:**
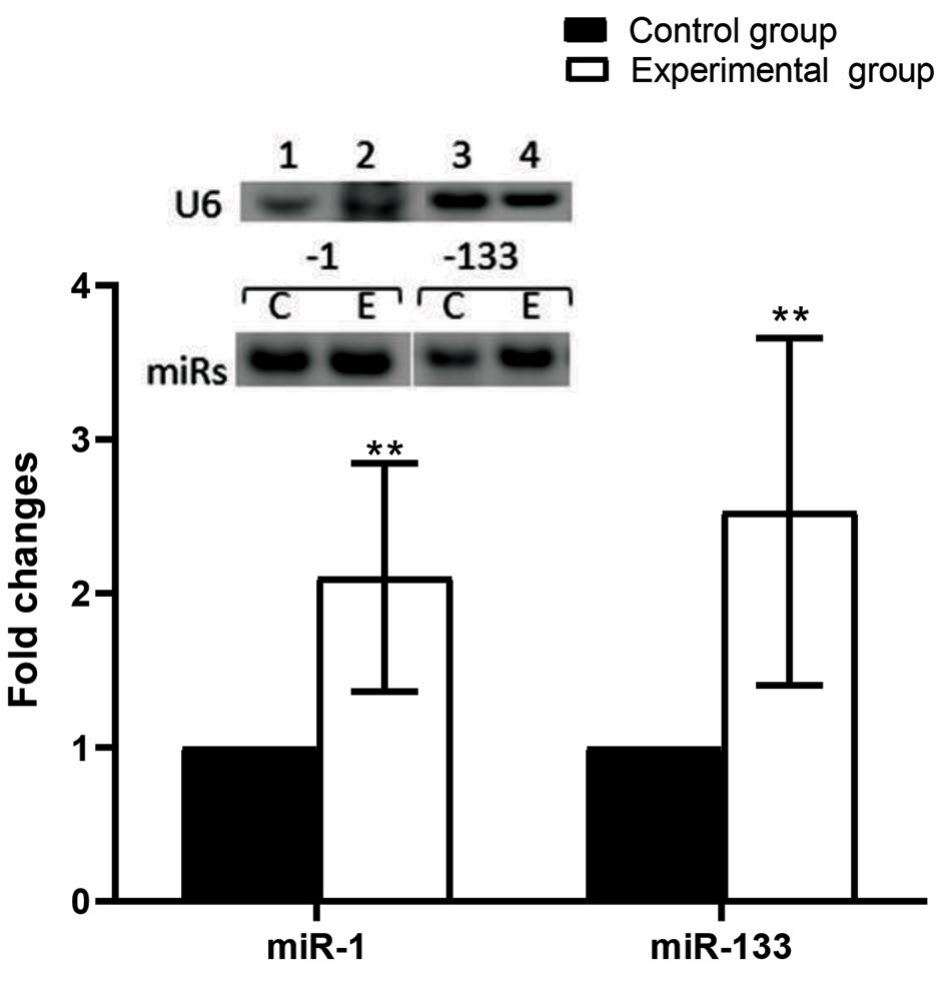
Expressions of miR-1 and miR-133 in the rats. Representative image of real-time polymerase chain reaction (PCR) results for miR-1 and miR- 133 in left ventricle tissue from the control (CON) and experimental groups (ET), respectively. The expressions of the microRNAs (miRNAs) were normalized to U6 expression. Histogram of real-time PCR miRNA products showed that both miR-1 and miR-133 had significantly increased expressions of greater than~2.1-fold (P=0.001 and P=0.004, respectively) in response to 14 weeks of endurance training). Values are mean ± SE (n=7). Data were analysed by the independent t test.∗∗; P<0.01.

The results of this study showed that the expressions of miR-1 (P=0.001) and miR-133 (P=0.004) in the left ventricle of the trained group significantly increased after 14 weeks of endurance training (Fig .3).

**Fig.4:**
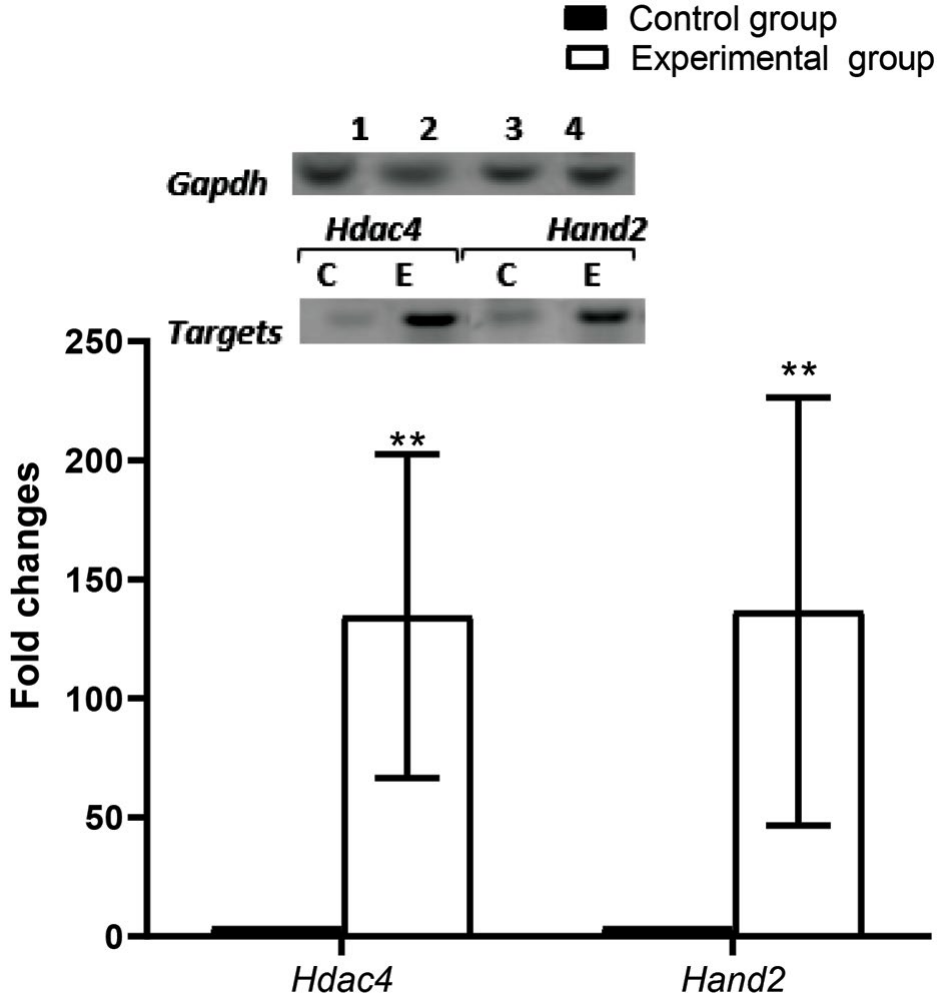
Expressions of histone deacetylase 4 (*Hdac4*) and heart and neural crest derivatives express 2 (*Hand2*) genes in rats. Representative image of real-time polymerase chain reaction (PCR) results for *Hdac4* and *Hand2* in left ventricle tissue from the control (CON) and experimental groups (ET), respectively. Expressions of the genes were normalized to glyceraldehyde-3-phosphate dehydrogenase (*GAPDH*) expression. Histogram of real-time PCR gene products revealed significantly increased expression of both genes by ~143-fold (P=0.001) in response to 14 weeks of endurance training. Values are mean ± SE (n=7). Data were analysed by the independent t test.∗∗; P<0.01.

**Fig.5:**
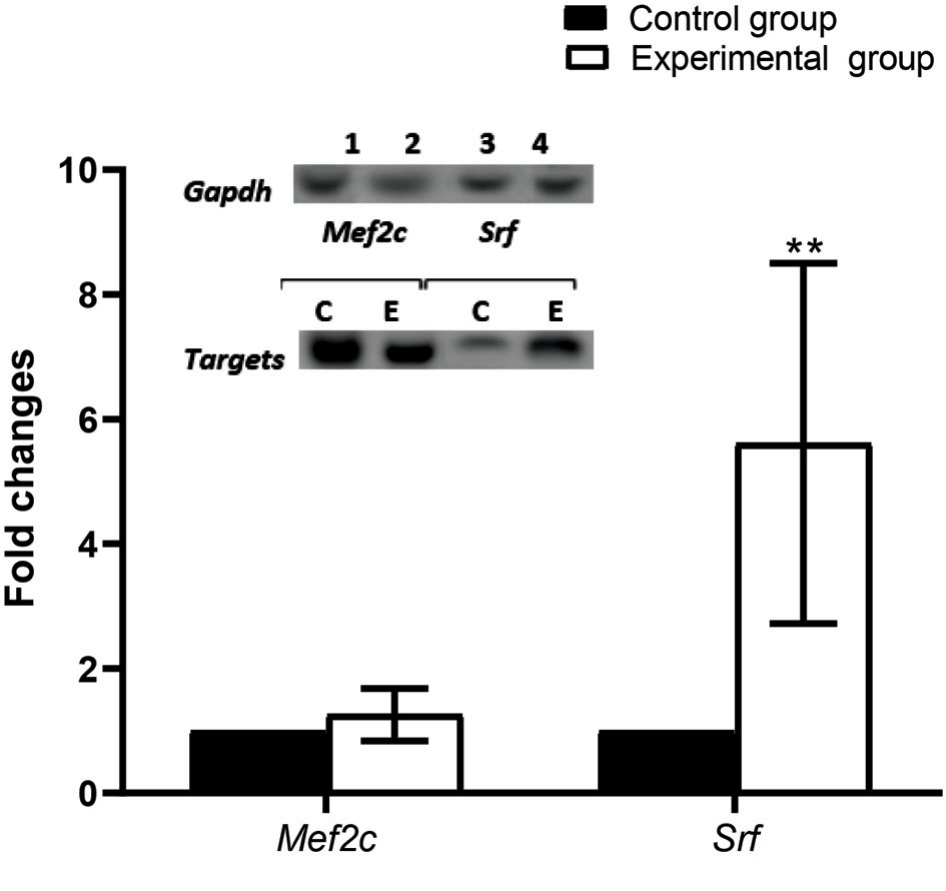
Expressions of *Mef2c* and *Srf* genes in the rats. Representative image of real-time polymerase chain reaction (PCR) results for *Mef2c* and *Srf* genes in left ventricle tissue from the control (CON) and experimental groups (ET), respectively. The gene expressions were normalized to glyceraldehyde- 3-phosphate dehydrogenase (*GAPDH*) expression. Histogram of real-time PCR gene products revealed that Srf expression was significantly increased by ∼5.9-fold (P=0.001) in response to 14 weeks of endurance training, whereas there was no difference in the expression level of *Mef2c* between the CON and ET groups (P=0.148). Values are mean ± SE (n=7). Data were analysed by the independent t test. **; P<0.01.

The expressions of *Hand2* and *Hdac4* genes in the left ventricle of the ET group rats significantly increased (P=0.001) by ~143-fold (Fig .4). *Srf* gene expression significantly increased (P=0.001) by ~5.9-fold in the left ventricle of the ET group rats after 14 weeks of ET There was no change in expression of the *Mef2c* gene (P=0.148, Fig .5).

## Discussion

The present study has demonstrated that 14 weeks of ET was able to change the expressions of the miRNAs and the genes that are part of a transcription network to regulate ventricular growth and cardiomyocyte differentiation of the heart, as has been described by van Rooij et al. (2).

Echocardiography and weighing were used to evaluate heart and left ventricle adaptation to ET. The aim of this study was to determine if the miR-1 and miR- 133 network expression changes and the accompanied genes that function to regulate ventricular growth and cardiomyocyte differentiation of the heart mediate ET induced-cardiac hypertrophy.

As in previous studies (21, 22), the results of this study showed that 14 weeks of ET-induced cardiac hypertrophy and improvement in some functional indexes. For example, LVW/BSA and LVW/BW in the experimental group were significantly more than the control group. These findings confirmed cardiac hypertrophy due to ET, as known physiological hypertrophy. The results of this study were in line with those of other studies (22, 23). The obtained data from echocardiography showed an increase in LVDd, which coincided with improved heart functional indexes (SV, FS%, and EF%). Similarly, several studies showed that ET increased SV, FS%, and EF% (24, 25). It is a possibility that the observed changes in heart structural and functional indexes in this study refer to physiological hypertrophy, which occurs without fibrosis and cardiac dysfunction. Aerobic exercise training, such as long-distance running or swimming, is matched with an increased volume overload accompanied by cardiac chamber dilation, and is referred to as eccentric hypertrophy (26). This phenotype is associated with the addition of sarcomeres in series to lengthen the cardiomyocytes and to increase the widths of the cells in parallel.

The interesting findings in this study was when the absolute weight of the heart and LV of the ET group compared with the CON group. There were no significant differences between the groups. In line with these findings, Pluim et al. (27), in a meta-analysis study, reported that the development of an endurance-trained heart (eccentric hypertrophy) and a strength-trained heart (concentric hypertrophy) was not to be considered an absolute and dichotomous concept, but rather a relative concept. However, when the weight of the heart and LV of the experimental group were assessed and compared to the ratio of BSA and BW of the rats, the differences were significant. Both HW/BW and LVW/BSA increased significantly in the experimental group.

As previous studies have pointed out (28, 29), physical activity, especially endurance activity, causes the heart to undergo cardiac remodelling (i.e., changes in left ventricular geometry) to enhance performance. The resulting phenotype is referred to as the “athlete’s heart” and is most frequently observed in elite athletes who participate in regular, high-intensity training regimes. A fundamental component of exercise-induced remodelling is physiological cardiac hypertrophy, a process that increases muscle mass by increasing cardiac myocyte size. Physiological cardiac hypertrophy is associated with normal or enhanced cardiac function (26). According to the mentioned studies, the structural and functional changes observed in this study were adaptations to ET and cardiovascular fitness to respond to the challenge of volume overload that occurred during ET.

The most important findings of this study were the increase of the miRNAs in proliferation and differentiation of myocardial cells such as miR-1 and miR-133 with their upstream and downstream genes (except for *Mef2c*).

Muscle-specific miR-1 and miR-133 are part of the myomiRs that play a key role in heart development and function. Both miR-1 and miR-133 are essential for cardiac remodelling in response to different stresses (30), including physical activity which induces physiological hypertrophy. In a clinical study, decreased expression of miR-133 was significantly associated with the severity of patients (31) since physical activity is a stimulator for cardiac remodelling and physiological hypertrophy; therefore, adaptation to this stimulant is necessary. Increases in miR-1 and miR-133 probably provide the base for physiological hypertrophy changes (32). According to a study by van Roij et al. (2), *Srf* and *Mef2c* are located upstream of miR-1 and miR-133 in DNA sequences. According to the result of this study, increased *Srf* expression probably induced increased miR-1 and miR- 133 expressions. However, in contrast to our results, Care and colleagues reported decreased expressions of cardiac miR-133 and miR-1 in pathological or physiological cardiac hypertrophy in rats and human pathological cardiac hypertrophy (13). The inconsistent results could be attributed to differences in the type of exercise. Our training protocol was ET and the Care protocol was an interval exercise. It seems the existing paradox could be due to the different types of exercise, because differences exist between interval and ET in the heart’s response to physical activity (22).

*Srf* is a transcription factor required for the regulation of important genes for cardiac structure and function. It has been reported that *Srf* is necessary for spontaneously induced hypertrophic gene expression in mouse cardiomyocytes (7). Probably, the increase in *Srf* gene expression is necessary for cardiac hypertrophy. However, some studies have pointed out that an increase in *Srf* occurs in pathological hypertrophy (33).

The *Mef2c* gene is involved in cardiac morphogenesis and myogenesis, and plays an important role in maintaining the differentiated state of muscle cells (34). Physical activity has been shown to increase *Mef2c* gene expression in rat trained muscles (35). *Mef2c* acts as a nodal point for stress-response and remodelling programs during cardiac hypertrophy and fibre-type switching muscles (36). On the other hand, it has been reported that physical activity induces fibre type transformation and cardiac hypertrophy, which coincide with mitochondrial biogenesis and other desirable adaptations. We only found one study in the field of exercise and cardiac adaptation. In contrast to our study results, Castro and colleagues reported unchanged and increased expression of cardiac *Mef2c* following medium intensity and high intensity swim training, respectively, in Atlantic salmon (37). It seems the differences are related to the differences in the subject or intensity of physical activity.

The result of this study showed that *Hdac4* gene expression dramatically increased after ET. *Hdac4* is a transcriptional repressor of muscle gene expression with chromatin remodelling. On the other hand, *Mef2c* activates miR-1 expression, which targets *Hdac4* and diminishes its repression (38). We observed increased *Hdac4* expression at the transcription level. However, the most important effects of *Hdac4* occur post-translation after physical activity (39), such as export of the Hdac4 protein from the cell nucleus (40) and phosphorylation of Hdac4 in response to exercise (39). Due to the lack of measurement of the Hdac4 protein, the discussion of increasing *Hdac4* depends on the Hdac4 protein measurement.

However, the important changes in heart (physiological, structural and functional) were measured in this study. We could not measure the protein levels of the miRNA downstream genes; therefore, these findings could not be generalized to protein changes. Additional studies are proposed to measure the level of proteins.

## Conclusion

After 14 weeks of ET, our research provided a comprehensive view of the network transcription factors of which miR-1 and miR-133 play a central role and support a relevant role of these myomiRs in the modulation of cardiac physiological hypertrophy. We also provided evidence for the cardiac functional and structural changes during ET that coincided with miR-1 and miR-133 changes, their upstream and downstream genes, and important elements in cardiac structural and functional changes.

## Supplementary PDF


