# Supplementary material for: The Changes of Heart miR-1 and miR-133 Expressions following Physiological Hypertrophy Due to Endurance Training
Source: Cell J. 2020 Sep 8;22(Suppl 1):133–40. doi: 10.22074/cellj.2020.7014 (PMC7481891; doi:10.22074/cellj.2020.7014)
Supplement: Supplementary file 1 [file Cell-J-22-Suppl1-133-s01.pdf]

www.celljournal.org

Supplementary Information for

The Changes of Heart miR-1 and miR-133 Expressions following Physiological Hypertrophy due to Endurance Training

Mohammad Fathi, Ph.D.[1]*, Reza Gharakhanlou, Ph.D.[2], Razieh Rezaei, Ph.D.[3]

1. Department of Physical Education and Sport Sciences, Faculty of Humanities Sciences, Lorestan University, Khorramabad, Iran
2. Department of Physical Education and Sport Sciences, Faculty of Humanities Sciences, Tarbiyat Modares University, Tehran, Iran
3. Faculty of Physical Education and Sport Sciences, Shahid Chamran University of Ahvaz, Ahvaz, Iran

***Corresponding Address: P.O.Box: 68151-44316, Department of Physical Education and Sport Sciences, Faculty of Humanity Sciences, Lorestan University, Khorramabad, Iran**
Email: Fathi.m@lu.ac.ir

Table S1: Primers used in the real-time polymerase chain reaction evaluation

Target	Primer sequence (5´-3´)	Tm	Size (bp)	Accession no.
GAPDH	F: AACCCATCACCATCTTCCAG	59.19	74	NM_017008.4
	R: CACGACATACTCAGCACCAG	58.37		
Mef2c	F: CCATTGGACTCACCAGACCT	58.01	84	XM_003749164.1
	R: ATGTTGCCCATCCTTCAGAG	57.57		
Hdac4	F: AACCTAACCTGAAATTACGGTC	60.36	137	NM_053449.1
	R: ACATGCGGAGTCTGTAACATC	60.11		
Hand2	F: CCAGCTACATCGCCTACCTC	59.68	163	NM_022696.1
	R: TTCTTGTCGTTGCTGCTCAC	59.06		
Srf	F: CACCTCCACAATCCAAACAG	56.62	89	NM_001109302.1
	R: GTGCCAGGTAGTTGGTGATG	57.55		
miR-133	205320 rno-miR-133a, LNA™ PCR primer set, UniRT .miRCURY LNA™ Universal RT microRNA PCR, microRNA primer set.			
miR-1	205104 rno-miR-1, LNA™ PCR primer set, UniRT .miRCURY LNA™ Universal RT microRNA PCR, microRNA primer set.			
U6	203907, U6 snRNA (hsa, mmu, rno) PCR primer set, UniRT. miRCURY LNA™ Universal RT microRNA PCR, reference gene primer set. NCBI Symbol U6snRNA, NCBI Accession: x59362			
